# Multiplex serum protein analysis reveals potential mechanisms and markers of response to hyperimmune caprine serum in systemic sclerosis

**DOI:** 10.1186/s13075-017-1252-x

**Published:** 2017-03-07

**Authors:** Niamh Quillinan, Kristina E. N. Clark, Bryan Youl, Jeffrey Vernes, Deirdre McIntosh, Syed Haq, Christopher P. Denton

**Affiliations:** 1 Centre for Rheumatology, UCL Division of Medicine, Royal Free Campus, Rowland Hill Street, London, NW3 2PF UK; 2 Department of Neurophysiology, Royal Free London NHS Foundation Trust, London, UK; 3 Daval International, London, UK

**Keywords:** Scleroderma, Clinical trial, Biomarker, Goat serum, Melanocortin

## Abstract

**Background:**

Hyperimmune caprine serum (HICS) is a novel biological therapy with potential benefit for skin in established diffuse cutaneous systemic sclerosis. Here we report multiplex protein analysis of blood samples from a placebo-controlled phase II clinical trial and explore mechanisms of action and markers of response.

**Methods:**

Patients were treated with HICS (*n* = 10) or placebo (*n* = 10) over 26 weeks, with follow-up open-label treatment to 52 weeks in 14 patients. Serum or plasma samples at baseline, 26 and 52 weeks were analysed using multiplex or individual immunoassays for 41 proteins. Patterns of change were analysed by clustering using Netwalker 1.0, Pearson coefficient and significance analysis of microarrays (SAM) correction.

**Results:**

Cluster analysis, SAM multiplex testing and paired comparison of individual analytes identified proteins that were upregulated or downregulated during treatment with HICS. There was upregulation of the hypothalamo-pituitary-adrenal axis after HICS treatment evidenced by increases in α-MSH and ACTH in cases treated with HICS. Interestingly, significant increase in PIIINP was associated with HICS treatment and improved MRSS suggesting that this may be a marker of extracellular matrix turnover. Other relevant factors reduced in HICS-treated patients compared with controls, although not reaching statistical significance included COMP, CCL2, IL6, TIMP2, Fractalkine and TGFβ1 levels.

**Conclusions:**

Our results suggest mechanisms of action for HICS, including upregulation of α-MSH, that has been shown to be anti-fibrotic in preclinical models, and possible markers to be included in future trials targeting skin in diffuse cutaneous systemic sclerosis.

**Trial registration:**

Eudract, No. 2007-003122-24. ClinTrials.gov, No. NCT00769028. Registered 7 October 2008.

**Electronic supplementary material:**

The online version of this article (doi:10.1186/s13075-017-1252-x) contains supplementary material, which is available to authorized users.

## Background

Hyperimmune caprine serum (HICS) has been reported to have beneficial effects in several disease settings with potential improvement in several neurological and inflammatory human diseases [1–5]. The mechanism of action in these conditions is poorly understood and likely multifactorial. Proposed pharmacodynamic effects of HICS include an effect on ion channel function [6–9], immunosuppression and neuroendocrine modulation through the hypothalamo-pituitary axis [10].

Recently reported clinical data from a phase II study in established diffuse cutaneous systemic sclerosis (dcSSc) suggest possible treatment benefit of HICS for skin over 26 weeks for active treatment compared with placebo and meaningful improvement in 50% of cases, and improvement in modified Rodnan skin score (MRSS) in an extended dataset including subjects that moved from placebo to active treatment after the first 26 weeks [11]. Here we report the effect of treatment on multiple serum proteins and examine the potential association between changes in analytes and modified Rodnan skin score in individual study subjects.

In addition to elucidating the effect of HICS, this systematic study of biologically relevant candidate molecular markers in a cohort of established dcSSc gives new insight to a phase of the disease that is associated with substantial morbidity and mortality, but that has often been excluded from clinical trials that generally focus on early diffuse systemic sclerosis (SSc). In this way we have highlighted the potential benefit of biological intervention/immunomodulation in established SSc and also demonstrated that information about skin treatment may be derived from this population.

## Methods

### Study cohort and clinical outcomes

This was a placebo-controlled study of 20 patients with established diffuse cutaneous SSc, defined by disease duration of at least 36 months from first non-Raynaud’s symptom. Subjects were randomly allocated 1:1 to active treatment or placebo. The clinical and demographic features of the study cohort have been described in detail previously [11]. Of note, three patients withdrew from the study; one in the HICS group due to cerebral infarction unrelated to study medication and two in the placebo group due to progression of disease. After 26 weeks of blinded treatment, all patients were offered open-label treatment for a further 26 weeks. The blind of original treatment allocation was maintained to 52 weeks. The flow of subjects for the study is shown in Additional file 1: Figure S1. For the primary analysis, baseline and 26-week MRSS were compared and statistical difference between the active treatment and placebo-treated cases was compared. Secondary analysis differentiated responders from non-responders defined by improvement in skin score by at least 20% of baseline and four MRSS units. Post hoc analysis of MRSS change included seven placebo cases that moved to the active treatment and three cases that continued without medication.

### Serum and plasma protein analysis

Blood samples were obtained at baseline, week 0 (pre- and post-injection of medication), weeks 26 and 52. Serum and plasma samples were sent frozen to Quest Diagnostics (Valencia, CA, USA) for single-protein analysis or Quansys Biosciences (Logan, UT, USA) for multiplex analysis, as outlined below.

Single-analysis assays were used for analysis of procollagen III N-terminal propeptide (PIIINP), soluble interleukin-2 receptor (sIL-2R), cartilage oligomeric matrix protein (COMP), transforming growth factor beta 1 (TGF-β1) and von Willebrand factor (vWF). vWF samples were plasma samples and an enzyme-linked immunosorbent assay (ELISA) (Cat. No. 84793; Aushon Biosystems, Inc., Billerica, MA, USA). Soluble IL-2R samples were serum samples and an ELISA (Cat. No. EH2IL2R; Thermo Fisher Scientific, Waltham, MA, USA). TGF-β1 samples were serum samples and an ELISA (Cat. No. DB100B; R&D Systems, Inc., Minneapolis, MN, USA). PIIINP samples were serum samples and a radioimmunoassay (Cat. No. 06098; Orion Diagnostica Ltd., Espoo, Finland).

Multiplex serum analysis was performed for αMSH, ACTH, ANG2, HGF, PDGF-bb, TIMP-1, TIMP-2, VEGF, FGF basic, Eotaxin, GRO-α, MCP-1, MCP-2, RANTES, I-309, TARC, IP-10, IL-1α, IL-1β, IL-2, IL-4, IL-6, IL-8, IL-10, IL-12p70, IL-13, IL-15, IL-17, IL-23, IFN-γ, TNF-α, TNF-β, IFN-α, IFN-β, Fractalkine and PARC by multiplex analysis. Samples were Quansys Biosciences by Q-Plex Array™ kits for Human Angiogenesis (No. 150251HU), Human Chemokine (No. 120251HU), and Human Cytokine (No. 110951HU). Both Fractalkine and PARC were custom developed from matched pair antibodies available from R&D Systems. The Q-Plex™ kits used in the sample testing have undergone extensive validation. Ranges for each assay were determined by dilutions determining upper ranges where high-end hook effect and apparent antibody saturation are avoided and lower ranges that are above detection limits. Antigen standard curves were performed in duplicate.

### Statistical analysis

For each analyte there was an individual comparison of baseline, 26 and 52 weeks and an integrated multiplex analysis to look for clusters of change in groups of cytokines that may reflect treatment with HICS or clinical differences in MRSS occurring during the study period. Unsupervised and supervised cluster analysis was undertaken to define baseline differences or changes over 26 and 52 weeks. Differences were compared between treatment groups and also for those with subgrouping strategies at baseline, longitudinally and linked to meaningful or numerical change in clinical variables. Permutation analysis was used to compare cytokine levels between the treatment arm and placebo arm. This was processed in EXCEL (Microsoft Corp., Redmond, WA, USA) and analysed using *t* tests with significance analysis of microarray (SAM) correction. Normalisation of data points and hierarchical clustering were performed using Netwalker 1.0 (http://netwalkersuite.org), and heat map construction was performed using CIMminer (https://discover.nci.nih.gov/cimminer/). Scatter plots were used to explore the association between MRSS and individual analytes. Baseline and 26-week values were compared between HICS and control treatment arms in the extended dataset and changes were also examined in the subset of subjects that changed from placebo to HICS at 26 weeks and for those that received HICS over a total of 52 weeks.

In order to normalise data, the analyte titre was divided by the average titre for all patients at time point zero, and then log transformed. The normalized data were expressed as a value that is centred on the mean level of the analyte across all the samples examined. This is a well-established and validated method which permits comparison of multiple proteins in each subject and allows the levels to be compared for different proteins within the cohort – this both vertical and horizontal clustering can be achieved for the heat maps. We have expanded the “Methods” section of the revised manuscript to explain normalization methods included in our analysis in more detail. When using SAM analysis, the cutoff of significance is determined by tuning the parameter delta, and then selection based on the fold change and q value. This highlighted genes that were differentially expressed. A combination of fold change and q value together with level of statistical significance in univariable comparison for HICS treatment effect compared with placebo were used to select hallmark upregulated or downregulated analytes that are included in Table 1 and annotated on heat maps.

**Table 1:** Representative serum analytes (mean [SD]) for subjects receiving HICS or placebo treatment over 26 weeks

	Change during study HICS			Change during study placebo			HICS versus placebo		
Direction of change	Basal	26 wk	26 wk versus basal *p* value	Basal	26 wk	26 wk versus basal *p* value	Fold change	q value	*p* value
Sample number	*n* = 10	*n* = 9	*n* = 9	*n* = 10	*n* = 10	*n* = 10			
UP									
α-MSH pg/ml	3.7 [3.6]	31.1 [35.8]	0.0035	1.8 [0.9]	2.2 [1.9]	0.75	299.6	0	0.039
ACTH pg/ml	1.9 [2.4]	27.6 [42.3]	0.0099	1.1 [1.1]	1.0 [0.7]	0.97	176.6	0	0.05
bFGF pg/ml	3.4 [6.5]	21.5 [21.9]	0.0185	21.3 [43.3]	23.6 [51.6]	0.62	65	0	0.15
PIIINP ug/ml	6.9 [3.8]	15.4 [10.1]	0.0002	5.3 [2.7]	5.9 [2.7]	0.62	14.6	20	0.012
DOWN									
COMP ng/ml	1.8 [1.1]	1.4 [0.3]	0.055	1.3 [0.5]	1.4 [0.6]	0.7	−3.38	36	0.265
FRACT (CX3CL1) ng/ml	3.7 [6.4	3.3 [6.1]	0.108	1.1 [0.7]	1.0 [0.6]	0.79	−6.1	43	0.318
CCL2 (MCP1) pg/ml	262.3 [101.1]	202.4 [132.0]	0.239	280.6 [133.6]	325.8 [225.9]	0.293	−0.5	36	0.121
TIMP2 ng/ml	13.4 [1.9]	13.0 [1.8]	0.322	13.1 [1.8]	13.9 [2.3]	0.172	−12.6	43	0.1044
TGFβ1 ng/ml	29.4 [7.9]	27.8 [4.3]	0.901	26.4 [4.4]	25.4 [10.4]	0.385	−3.8	43	0.601
Gro-α pg/ml	35.0 [97.2]	10.7 [17.1]	0.076	3.6 [5.5]	10.2 [15.1]	0.939	−2.3	36	0.206
IL6 pg/ml	4.6 [9.7]	1.4 [1.4]	0.309	25.4 [69.5]	30.6 [66.8]	0.128	−0.04	36	0.053

*SD* standard deviation, *HICS* hyperimmune caprine serum

## Results

### Clinical outcome data for modified Rodnan skin score

**Fig. 1:**
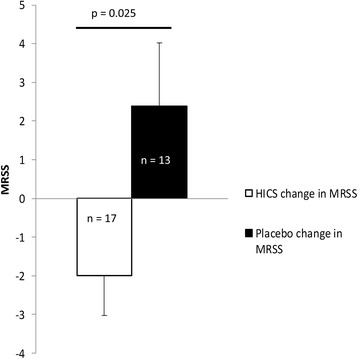
Change in modified Rodnan skin score after 26 weeks of treatment with hyperimmune caprine serum compared with placebo or no treatment. In the extended clinical trial dataset patients received 26 weeks of therapy with HICS or placebo. Seven subjects that had received placebo were then treated with HICS. For all subjects there was a 52-week visit and so MRSS between 26 and 52 weeks was available for placebo-treated cases, providing an extended dataset of 17 subjects receiving HICS and 13 with no active treatment. Treatment blind for the first 26 weeks was maintained to week 52. There was statistically significant difference between these two treatment groups. These data extended the dataset from the placebo-controlled phase of the study that had demonstrated a trend of improvement for MRSS and for responder frequency defined by improvement of at least four skin score units and 20% of baseline MRSS. The placebo-controlled phase has been previously described [11]. *HICS* hyperimmune caprine serum, *MRSS* modified Rodnan skin score

This manuscript focuses on pre-specified analysis of multiple serum proteins over 52 weeks. For the first 26 weeks, the enrolled subjects (*n* = 20) were randomly assigned to self-administer twice weekly HICS by subcutaneous injection or matched placebo. Following this period, all subjects were offered the opportunity to receive 26 weeks of active HICS treatment. Three of the study cohort did not elect to receive HICS during this second 26-week period. The primary efficacy end point for this trial was change in MRSS measured as a continuous variable and comparing baseline and 26-week values. The clinical data for the placebo-controlled trial have been previously reported [11]. Briefly, mean mRSS fell by 1.4 ± 4.7 units with active treatment but worsened by 2.1 ± 6.4 units on placebo (*p* = 0.181, unpaired *t* test) when baseline values were compared with 26-week values. Responder analysis showed that 50% of HICS patients improved compared to 10% of placebo patients at 26 weeks (*p* = 0.062). We undertook further analysis of an extended dataset, comparing placebo patients who took medication on a compassionate basis after the double-blind phase of treatment for a further 26 weeks. Thus, skin score data were available for seven additional cases treated for 26 weeks with HICS, and from three cases that chose not to take the drug but that were observed for a further 26 weeks off treatment. Skin score data for this extended dataset comprising 13 subjects receiving placebo or no active therapy with the 17 subjects that received HICS for 26 weeks confirms statistically significant difference between active treatment and controls in a post hoc analysis, *p* = 0.025 (Fig. 1).

### Serum and plasma protein analysis

The data for the 26-week placebo-controlled phase of the study for PIIINP, vWF and sIL2R have been presented previously [11]. Here we show additional data to week 52 that confirm the earlier findings (Table 1). For the baseline measurements, there were 20 subjects, at 26 weeks there were *n* = 10 receiving placebo and *n* = 9 receiving HICS due to one patient discontinuing treatment early in the study. Fifty-two-week follow-up was on a compassionate basis with safety review and so fewer samples were available. For clarification, the total study cohort was 20 subjects. There were samples available for assay from *n* = 7 subjects who had moved from placebo to HICS, *n* = 6 subjects continuing HICS treatment and *n* = 2 subjects who moved from HICS to no treatment and *n* = 3 subjects who were in the placebo arm and chose not to receive HICS from weeks 26 to 52. The flow of patients through the study is illustrated in the schematic in Additional file 1: Figure S1.

### Cluster analysis and heat maps

**Fig. 2:**
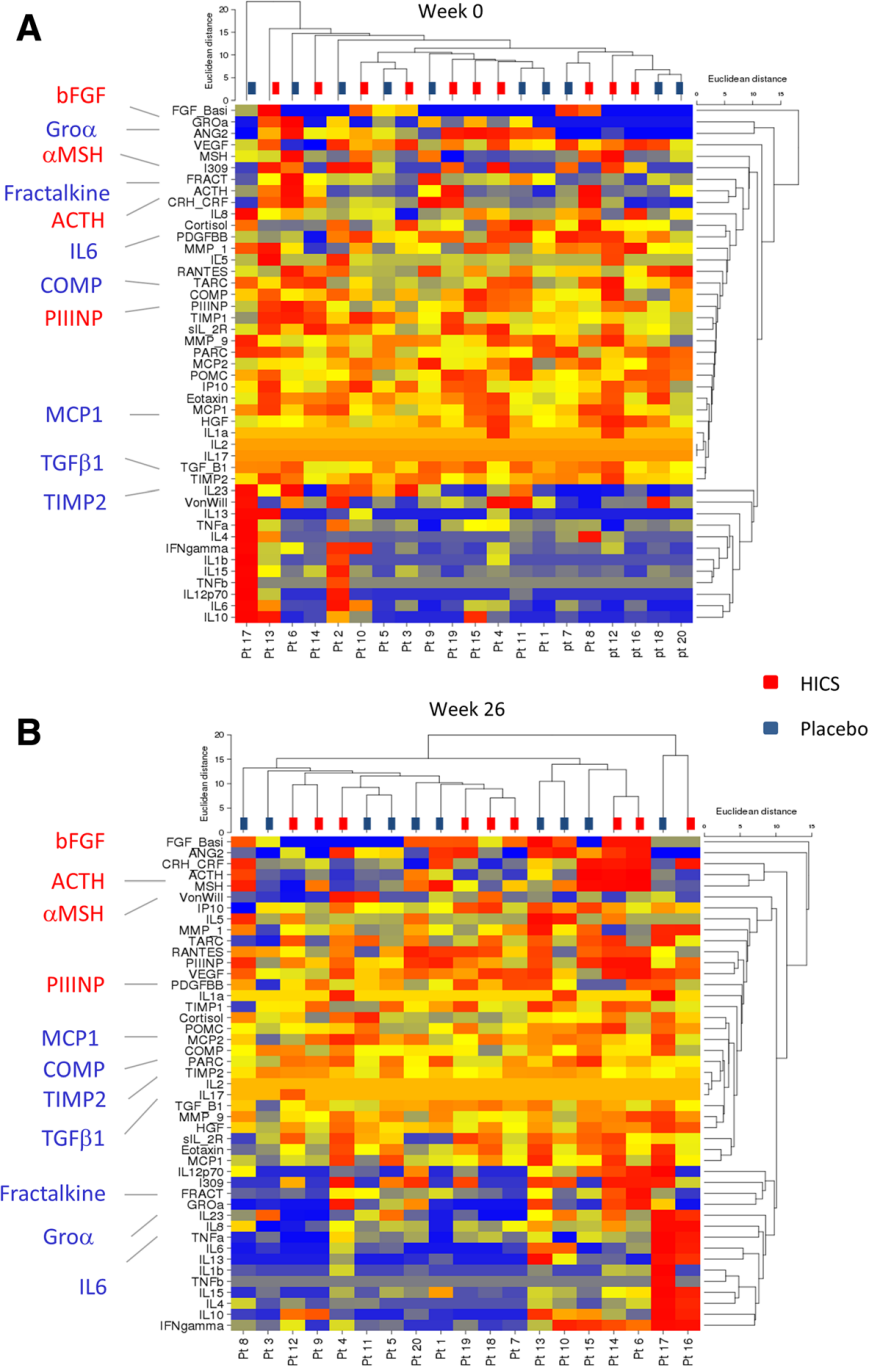
Unsupervised hierarchical cluster analysis for multiplex serum proteins at baseline and 26 weeks during the placebo-controlled trial of HICS. Unsupervised cluster analysis was undertaken to identify any subgroups within the study cohort at baseline based upon the serum levels of multiple protein analytes as described in text. The same analysis was repeated for serum samples after 26 weeks of treatment with HICS or placebo. The randomly assigned treatment allocation is shown for each subject. Data for baseline samples are shown in panel **a**. After 26 weeks of treatment there were clear changes in the patterns of protein analytes that were spread between the two treatment arms as shown in panel **b**. *HICS* hyperimmune caprine serum

**Fig. 3:**
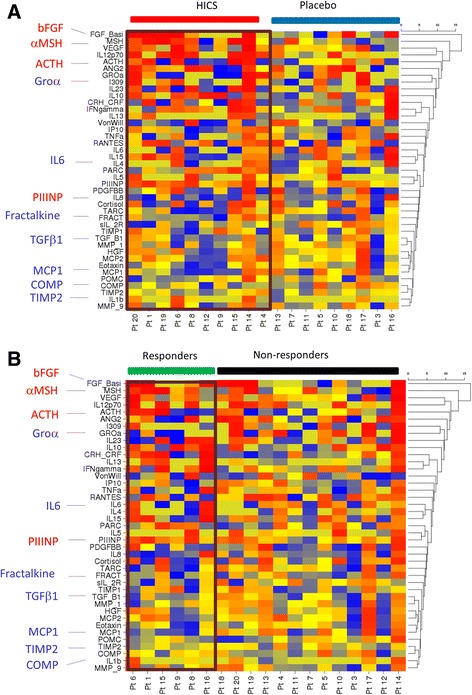
Supervised analysis for change in serum proteins according to treatment or responder status in the 26-week placebo-controlled trial of HICS. **a** Cluster analysis was undertaken for serum analytes after allocation to HICS or placebo during the 26-week parallel-group controlled phase of the clinical trial to identify any subgroups within the study cohort at baseline based upon the serum levels of multiple protein analytes as described in text. A pattern of upregulation or downregulation of specific proteins is seen more frequently in cases treated with HICS than controls. **b** The same analysis was repeated with subjects allocated to responder or non-responder categories based upon improvement in MRSS by at least four skin score units and 20% of baseline MRSS. Of the responders 5/6 were in the HICS-treated arm of the study. In this way groups of proteins that are clustered for similar patterns of change in response to HICS or associated with clinically meaningful improvement in MRSS

First, the dataset was examined using cluster analysis with generation of heat maps to identify differences in the protein levels for the study cohort at baseline or 26 weeks. Unsupervised analysis of absolute concentrations, scaled and normalized for the purposes of comparison, did not show any clear differences for baseline values (Fig. 2a) whilst the 26-week data are more clustered (Fig. 2b). Changes were examined to explore the potential effect of HICS on serum protein levels of the large number of analytes and specifically focus on those cases that had shown a clinically meaningful improvement in MRSS (responders) compared to those that had stable or worsening skin score. Unsupervised clustering was first performed (Additional file 2: Figure S2) and later supervised analysis (Fig. 3). For these analyses the primary exploration was for data from the first placebo-controlled phase of the study, but the extended dataset including the additional seven patients treated for the first time with HICS over weeks 26 to 52 are also included as a secondary analysis to confirm and extend the data from the placebo-controlled phase. These are included in Additional file 3: Figure S3.

To provide a clearer signal of potential serum protein changes seen in the SSc subjects receiving active treatments in the placebo-controlled phase of the study, a summary heat map was generated for the placebo-controlled phase of the study. This demonstrates that some markers are shared and these may be markers of clinical change or response. In contrast the signature comparing HICS and placebo (Additional file 4: Figure S4) is more likely to be a direct effect of the HICS administration and may therefore be a better reflection of the impact of this novel therapy rather than the clinical changes occurring in MRSS, that might also provide insight into potential markers of change in skin severity that may be independent of treatment effect (Additional file 4: Figure S4). For the key analytes highlighted in Table 1 the heat maps are annotated showing that for almost all of these the change seen with HICS treatment compared with placebo was also observed in analysis based upon the responder status for MRSS.

### Statistical analysis of changes in serum proteins associated with HICS treatment

We next undertook statistical analysis of the whole dataset using SAM. The strength of this methodology is that it takes account of the multiple simultaneous analyses that are performed and corrects the data for false discovery risk. From this analysis a signature of proteins up- or downregulated by HICS emerged and these proteins were then subjected to a more detailed analysis of change over the 52 weeks of the study. These proteins are shown in Table 1 together with the basal and 26-week level, fold change and significance assessed by SAM and for individual statistical analysis of the analytes.

**Fig. 4:**
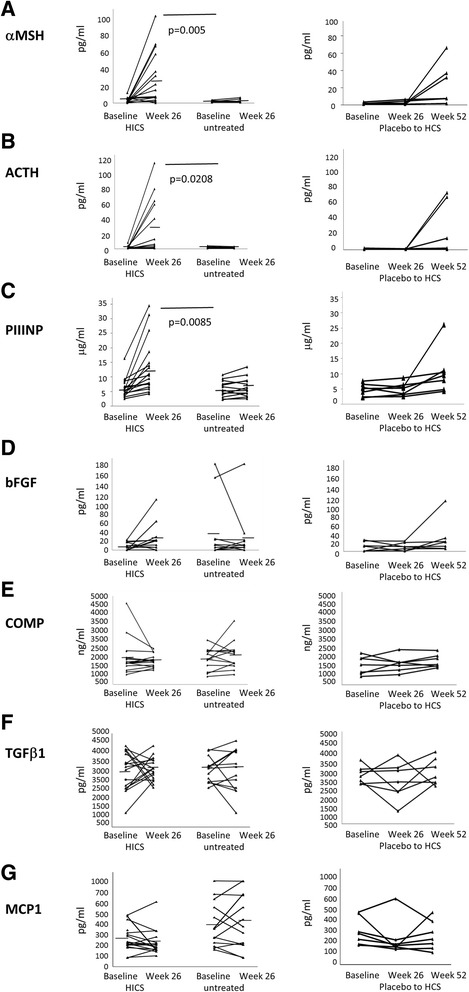
Baseline MRSS correlation and change during treatment with HICS for selected proteins. Individual patient data is shown for key serum proteins identified as significantly increasing during the 26-week treatment phase of the placebo-controlled trial. The panels compare changes in the HICS-treated or control extended dataset over 26 weeks that extend the data included in Table 1 for the placebo-controlled phase of the study. Adjacent plots show the change in the extended 52-week dataset for cases that moved from placebo to HICS at 26 weeks. These findings confirm the clear pattern of upregulation for PIIINP (**a**), αMSH (**b**), and ACTH (**c**). There is a trend for increase in bFGF (**d**) whilst the mean levels for COMP (**e**), TGFβ1 (**f**), and MCP1 (**g**) shows a fall over 26 weeks with HICS treatment. Changes in these key analytes also demonstrate the heterogeneity of the groups consistent with the heat maps shown above, with some subjects being obvious outliers and only some cases showing increase in serum levels after moving from placebo to HICS treatment at 26 weeks. *HICS* hyperimmune caprine serum

To further demonstrate the changes in proteins that are upregulated by HICS at 26 weeks, the change in cases that switched from placebo to active treatment between weeks 26 and 52 treatment effect individual patient data are plotted in Fig. 4 for key proteins included in Table 1. Congruity of mean and median values suggests near normal distribution and non-parametric assessment and the results are not significantly different from the methods included in the current manuscript. As detailed below to avoid confusion and ensure consistency with the external statistical analysis included in our final study report, we prefer to keep the results of parametric testing in this manuscript. The most significant changes were observed for αMSH, ACTH, and PIIINP. This demonstrates patterns of change in the extended dataset and in subjects moving from placebo to active treatment at 26 weeks and those receiving HICS over a 52-week period. For these analytes, individual subjects show clear evidence of increase similar to that seen over 24 weeks for actively treated subjects. Although the small numbers preclude formal statistical analysis, the data provide support to the effect seen in the first 26 weeks of the study and in the extended dataset. Mean and median values of relevant serum proteins, including COMP, CCL2, IL6, TIMP2, Fractalkine and TGFβ1were reduced in HICS-treated patients compared with controls, although not reaching statistical significance, and are shown in Table 1.

At an individual patient level levels of some analytes, including COMP, TGFβ1, αMSH and PIIINP that showed significant correlation for baseline MRSS confirming previous published data from other independent studies. However, there was no correlation between changes in these analytes and MRSS change during the study at an individual patient level (data not shown).

## Discussion

In this study we demonstrate the value of including biological analyses exploring potential markers and mechanisms of treatment effect in clinical trials of novel potential therapeutics, such as hyperimmune caprine serum [12]. By including later stage dcSSc we highlight the feasibility of recruiting this subgroup that may have some advantages (e.g. clinical homogeneity, acceptability of requiring no other immunosuppression and acceptability of a true placebo control) compared with early-stage SSc [13]. It is notable that there was progression in the placebo group in this study that is contrary to recent suggestions that later stage dcSSc is likely to be in a regressive stage [14]. A potential explanation for this is that immunosuppressive treatments were withdrawn in the study subjects and this may have led to more active skin disease.

Simultaneous measurement of multiple analytes is a powerful approach that has been applied in cross-sectional analyses but few longitudinal studies [15, 16]. We identify a molecular signature of HICS response in SSc and correlate with potential treatment effect that includes proteins that increase with HICS treatment compared with untreated cases, especially PIIINP, αMSH and ACTH, and also proteins that showed trends of reduction in the HICS-treated subjects. Our findings suggest potential for development of composite serum biomarkers for HICS response in SSc and could inform development of a more generic composite serum biomarker for SSc [17–19]. This would complement other markers that include serum variables and tests of other available markers such as the enhanced liver fibrosis (ELF) test [20] and confirm the feasibility of this approach. Our study has the particular strength of having longitudinal sampling and simultaneous assessment of MRSS. The changes observed for HICS treatment are notable in the context of falling MRSS. For PIIINP, the changes are unexpected in that there is increase in the context of improved MRSS both in the overall treatment cohort for HICS and also in the responder analysis (Additional file 3: Figure S3). This suggests that PIIINP may be a marker of extracellular matrix (ECM) remodeling as well as fibrotic burden as discussed below. Other proteins showing significant change include markers of activation (αMSH and ACTH) with highly significant increase at 26 weeks suggesting sustained upregulation of the hypothalamic pituitary axis in response to HICS. COMP has previously been suggested as a marker of skin score in scleroderma and so a trend for reduction in HICS-treated cases but not controls in notable.

Several distinct possible effects of HICS that have previously been suggested are highly relevant to the study. First, HICS is likely to have an immunomodulatory effect. This may reflect the presence of proteins and cytokines that affect immune function [21], including polyclonal immunoglobulin that has previously been suggested to be beneficial for some aspects of SSc. A recent retrospective single-centre observational study of 30 patients with refractive dcSSc receiving intravenous immunoglobulin (IVIG) showed significant reduction in MRSS at 24 months, indicating that it may be an effective adjunctive treatment [22]. To date, only one randomised double-blind trial has been completed. In this trial, a single 5-day course of IVIG did not show significant improvement but a retreatment with a second course showed an improvement in skin score [23]. Other recent reports suggest benefits in gastrointestinal symptoms and myositis in SSc patients receiving IVIG [24, 25].

One of the most compelling potential anti-fibrotic mechanisms for HICS is through stimulation of the hypothalmo-pituitary axis. It is notable that there is considerable evidence that stimulation of MSH pathways may benefit preclinical animal models of fibrosis and in vitro studies with human tissue [26–28]. For example, Bohm et al. described that human dermal fibroblasts express the MC1 receptor (MC1R) that binds α-MSH with high affinity and they found that α-MSH suppressed TGF-β-induced collagen synthesis in vitro [29, 30]. Furthermore, the authors used a bleomycin mouse model to investigate the effects of α-MSH on skin fibrosis and found that simultaneous administration of α-MSH with bleomycin suppressed the effects of bleomycin on HDF. ACTH was also found to have similar suppressive effects. α-MSH exerts its effects via a cAMP-driven pathway and not via Smad 2/3. α-MSH upregulates superoxide dismutase 2 and hemeoxygenase 1, which is protective against the effects of bleomycin on reactive oxygen species. They also confirmed the presence of POMC and the MC1R in affected skin from patients with SSc and dermal fibroblasts strongly expressed both POMC and MC1R [31]. In a recent study MC1-signalling-deficient mice were susceptible to bleomycin-induced fibrosis, whereas wild-type animals were not [32].

Strengths of this work include prospective definition of clinical phenotype, collection within the framework of a double-blind clinical trial and standardised assay methodology. Longitudinal sampling of candidate makers and placebo control data over a clinically meaningful duration allows more robust interpretation than in cross-sectional studies. There are also some limitations such as the relatively small number of study subjects in a heterogeneous disease so that subgroups may be distributed unevenly between the treatment groups. The need to use different platforms for some single-factor assays may be a limitation compared with using the same methods for all analyses. In addition, conclusions may only apply to late-stage diffuse cutaneous SSc. It would be useful in future work to extend this and include other stages and subsets of SSc such as limited cutaneous disease (lcSSc) or early diffuse SSc. The effect of previous immunosuppression cannot be reliably explored in this study but interplay between HICS and other more conventional immunomodulatory approaches that are in use as treatment for SSc or its complications could be addressed in future work.

## Conclusions

These findings provide important information about future potential for SSc molecular markers. They provide important insight into the potential biological effects of HICS in SSc and in other medical indications. The data are hypothesis generating and suggest potential future therapeutic avenues to explore such as the effect of HICS in a larger and more diverse clinical cohort, and possible development of new composite serum markers that reflect changes in MRSS in clinical trials across the disease spectrum.

## Additional files

Additional file 1: Figure S1. Subject progression in the clinical trials with serum sample time points. Schematic indicates how 20 subjects were randomly allocated to active treatment with hyperimmune caprine serum (HICS) or placebo. One patient withdrew from the HICS arm and was not available for follow-up. Other subjects were all followed to 52 weeks and at 26 weeks were offered open-label compassionate HICS. The study blind for 0–26 week treatment was maintained until after 52-week assessment. Serum and plasma samples were available for weeks 0, 26 and 52. Additional screening blood samples (pre-randomisation) were available for quality control purposes. (TIF 132 kb)
Additional file 2: Figure S2. Unsupervised hierarchical cluster analysis for multiplex serum proteins at baseline and 26 weeks for the extended dataset of 26 weeks treatment with HICS. Unsupervised cluster analysis was undertaken to identify any subgroups within the extended dataset of the study cohort at baseline, the end of placebo treatment period, or at 52 weeks based upon the serum levels of multiple protein analytes as described in text. This provided a larger sample size by including 17 subjects treated with HICS over 26 weeks and 13 subjects with 26 weeks of observation on placebo or no active treatment. The same analysis was repeated for serum samples after 26 weeks of treatment with HICS or placebo. The randomly assigned treatment allocation is shown for each subject. Data for baseline samples are shown in panel A together with treatment allocation. After 26 weeks of treatment there were clear changes in the patterns of protein analytes that were spread between the two treatment arms as shown in panel B. (TIF 1373 kb)
Additional file 3: Figure S3. Unsupervised cluster analysis for change in serum proteins from baseline to 26 weeks comparing treatment with hyperimmune caprine serum (HICS) with placebo over 26 weeks and in extended dataset at 52 weeks. (A) Unsupervised cluster analysis of change in protein level during 26-week treatment phase of placebo-controlled trial reveals patterns of change that are reflected in the supervised analysis shown in Fig. 3. Thus, subjects receiving placebo show generally less treatment effect and those treated with HICS show the patterns consistent with the summary changes shown above. (B) Unsupervised cluster analysis is also performed for the extended 52-week dataset that includes subjects moving from placebo to active treatment (*n* = 7) in the second 26 weeks and three cases that have no active treatment and were previously on placebo. This complements the presentation of data for baseline and 26 weeks presented in Additional file 1: Figure S1 and shows close congruity for the two 26-week unsupervised heat maps in the extended dataset. (TIF 1444 kb)
Additional file 4: Figure S4. Average change in serum proteins comparing treatment with hyperimmune caprine serum (HICS) or placebo and for MRSS responders versus non-responder at 26 weeks. (A) Average change in serum protein was calculated for each treatment arm over 26 weeks and ranked according to fold change average after HICS treatment. (B) Similar analysis was undertaken for average protein changes in the subjects showing significant improvement of four skin score units and 20% of baseline MRSS score during the trial (responders) or these that did not demonstrate clinical response. These were ranked for the most increased proteins in responder cases. Key proteins that emerged as upregulated (*red*) or downregulated (*blue*) for HICS treatment, shown in Fig. 4, are annotated with *asterisks*. (TIF 761 kb)

## Abbreviations

COMP: cartilage oligomeric matrix protein
dcSSc: Diffuse cutaneous systemic sclerosis
ECM: Extracellular matrix
ELISA: Enzyme-linked immunosorbent assay
HICS: Hyperimmune caprine serum
IVIG: Intravenous immunoglobulin
MRSS: Modified Rodnan skin score
PIIINP: Procollagen III N-terminal propeptide
SAM: Significance analysis of microarray
SSc: Systemic sclerosis
TGF-β1: Transforming growth factor beta 1
vWF: von Willebrand factor
