# Supplementary figures and images for: Multiplex serum protein analysis reveals potential mechanisms and markers of response to hyperimmune caprine serum in systemic sclerosis
Source: Arthritis Res Ther. 2017 Mar 7;19:45. doi: 10.1186/s13075-017-1252-x (PMC5341430; doi:10.1186/s13075-017-1252-x)

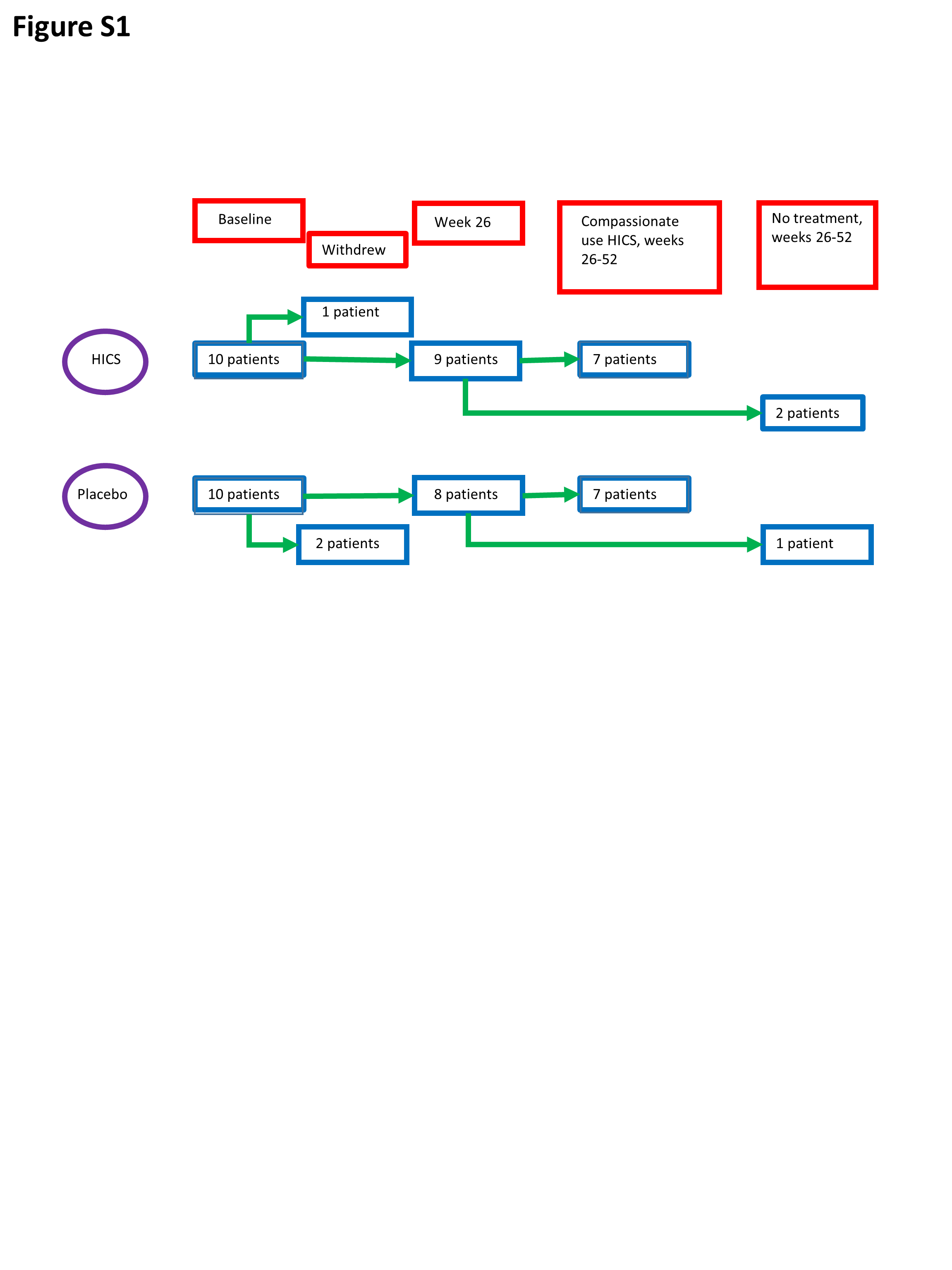

Supplement: Additional file 1: Figure S1. — Subject progression in the clinical trials with serum sample time points. Schematic indicates how 20 subjects were randomly allocated to active treatment with hyperimmune caprine serum (HICS) or placebo. One patient withdrew from the HICS arm and was not available for follow-up. Other subjects were all followed to 52 weeks and at 26 weeks were offered open-label compassionate HICS. The study blind for 0–26 week treatment was maintained until after 52-week assessment. Serum and plasma samples were available for weeks 0, 26 and 52. Additional screening blood samples (pre-randomisation) were available for quality control purposes. (TIF 132 kb) [file 13075_2017_1252_MOESM1_ESM.tif]

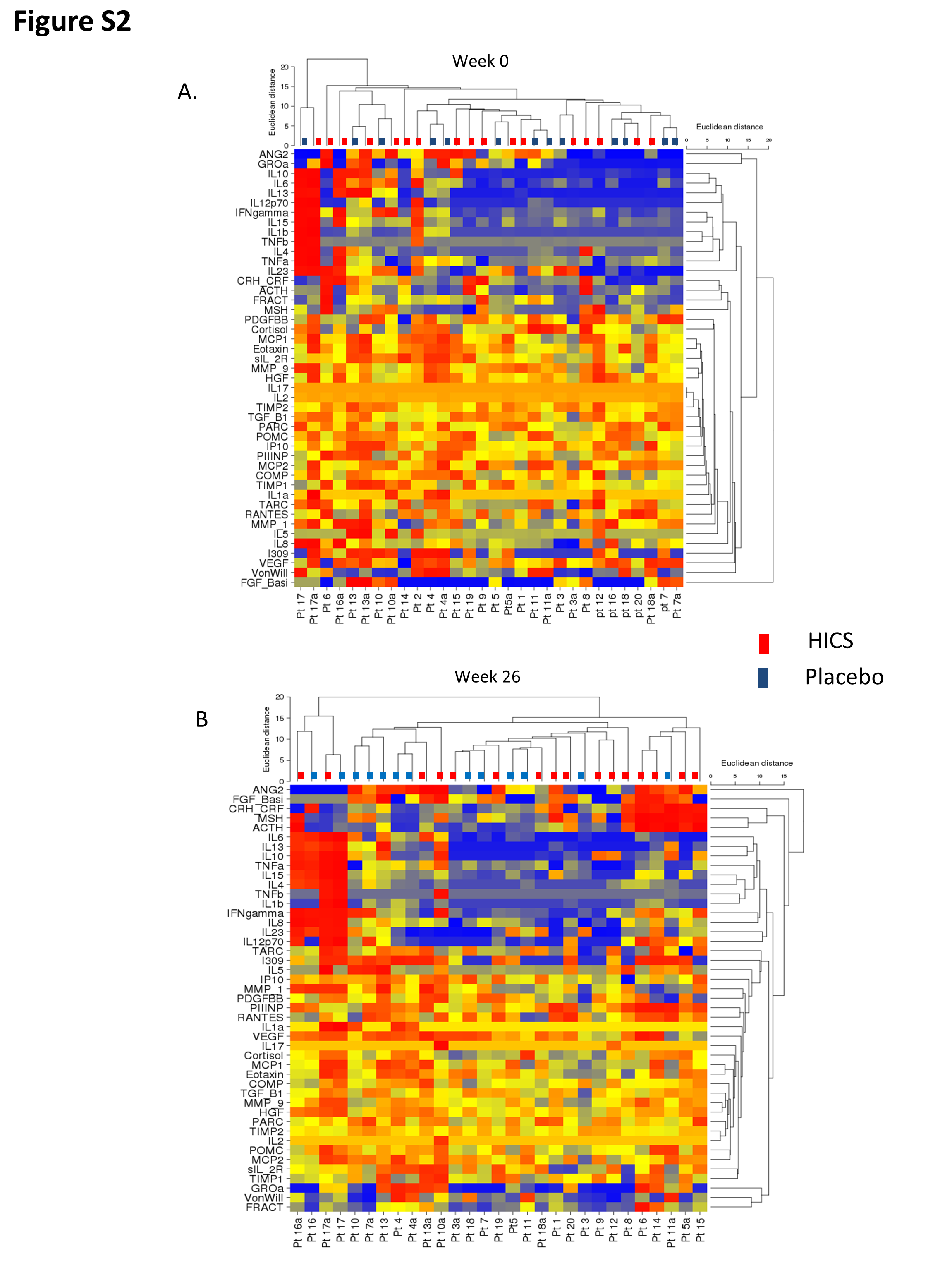

Supplement: Additional file 2: Figure S2. — Unsupervised hierarchical cluster analysis for multiplex serum proteins at baseline and 26 weeks for the extended dataset of 26 weeks treatment with HICS. Unsupervised cluster analysis was undertaken to identify any subgroups within the extended dataset of the study cohort at baseline, the end of placebo treatment period, or at 52 weeks based upon the serum levels of multiple protein analytes as described in text. This provided a larger sample size by including 17 subjects treated with HICS over 26 weeks and 13 subjects with 26 weeks of observation on placebo or no active treatment. The same analysis was repeated for serum samples after 26 weeks of treatment with HICS or placebo. The randomly assigned treatment allocation is shown for each subject. Data for baseline samples are shown in panel A together with treatment allocation. After 26 weeks of treatment there were clear changes in the patterns of protein analytes that were spread between the two treatment arms as shown in panel B. (TIF 1373 kb) [file 13075_2017_1252_MOESM2_ESM.tif]

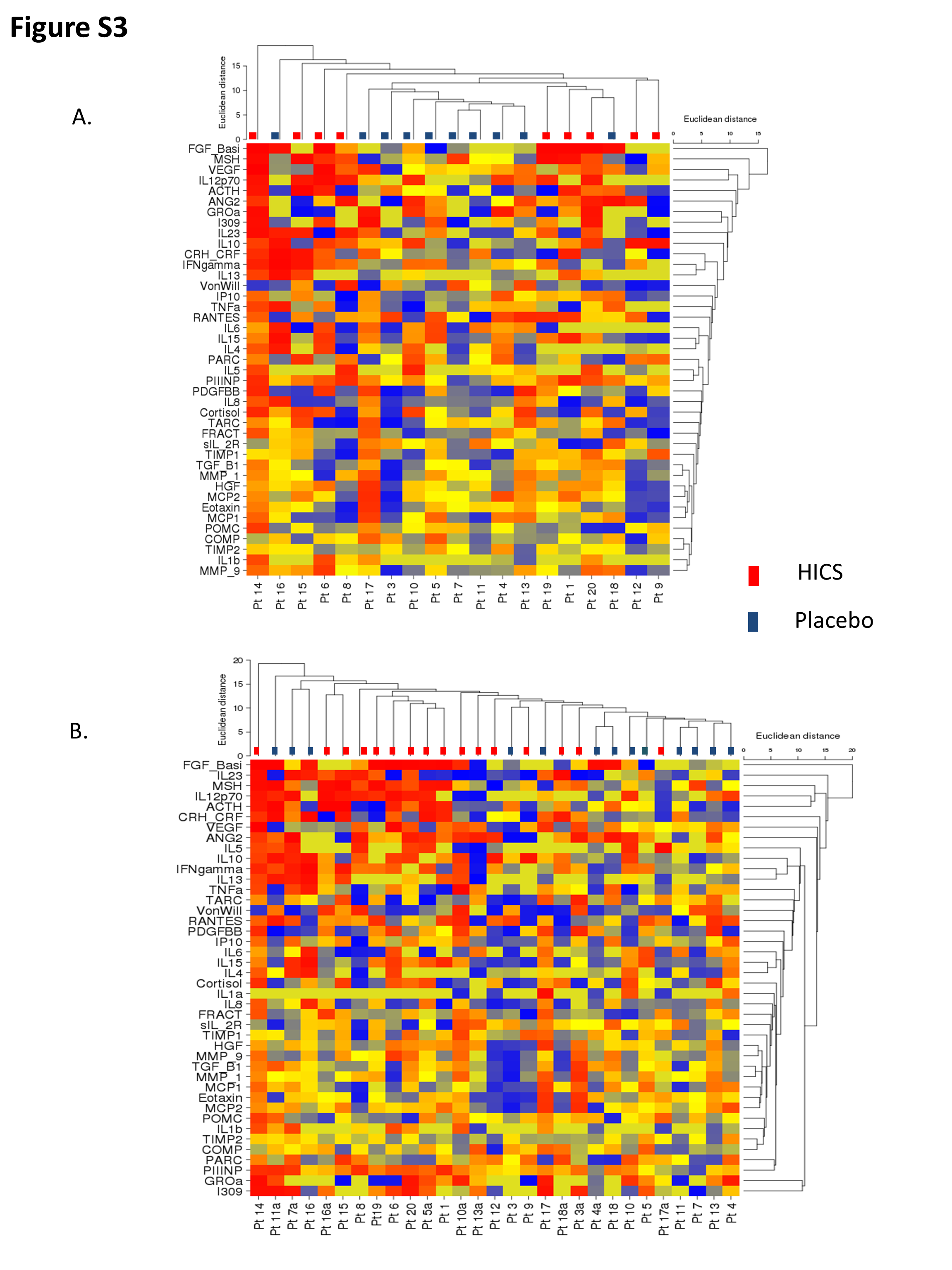

Supplement: Additional file 3: Figure S3. — Unsupervised cluster analysis for change in serum proteins from baseline to 26 weeks comparing treatment with hyperimmune caprine serum (HICS) with placebo over 26 weeks and in extended dataset at 52 weeks. (A) Unsupervised cluster analysis of change in protein level during 26-week treatment phase of placebo-controlled trial reveals patterns of change that are reflected in the supervised analysis shown in Fig. 3. Thus, subjects receiving placebo show generally less treatment effect and those treated with HICS show the patterns consistent with the summary changes shown above. (B) Unsupervised cluster analysis is also performed for the extended 52-week dataset that includes subjects moving from placebo to active treatment (n = 7) in the second 26 weeks and three cases that have no active treatment and were previously on placebo. This complements the presentation of data for baseline and 26 weeks presented in Additional file 1: Figure S1 and shows close congruity for the two 26-week unsupervised heat maps in the extended dataset. (TIF 1444 kb) [file 13075_2017_1252_MOESM3_ESM.tif]

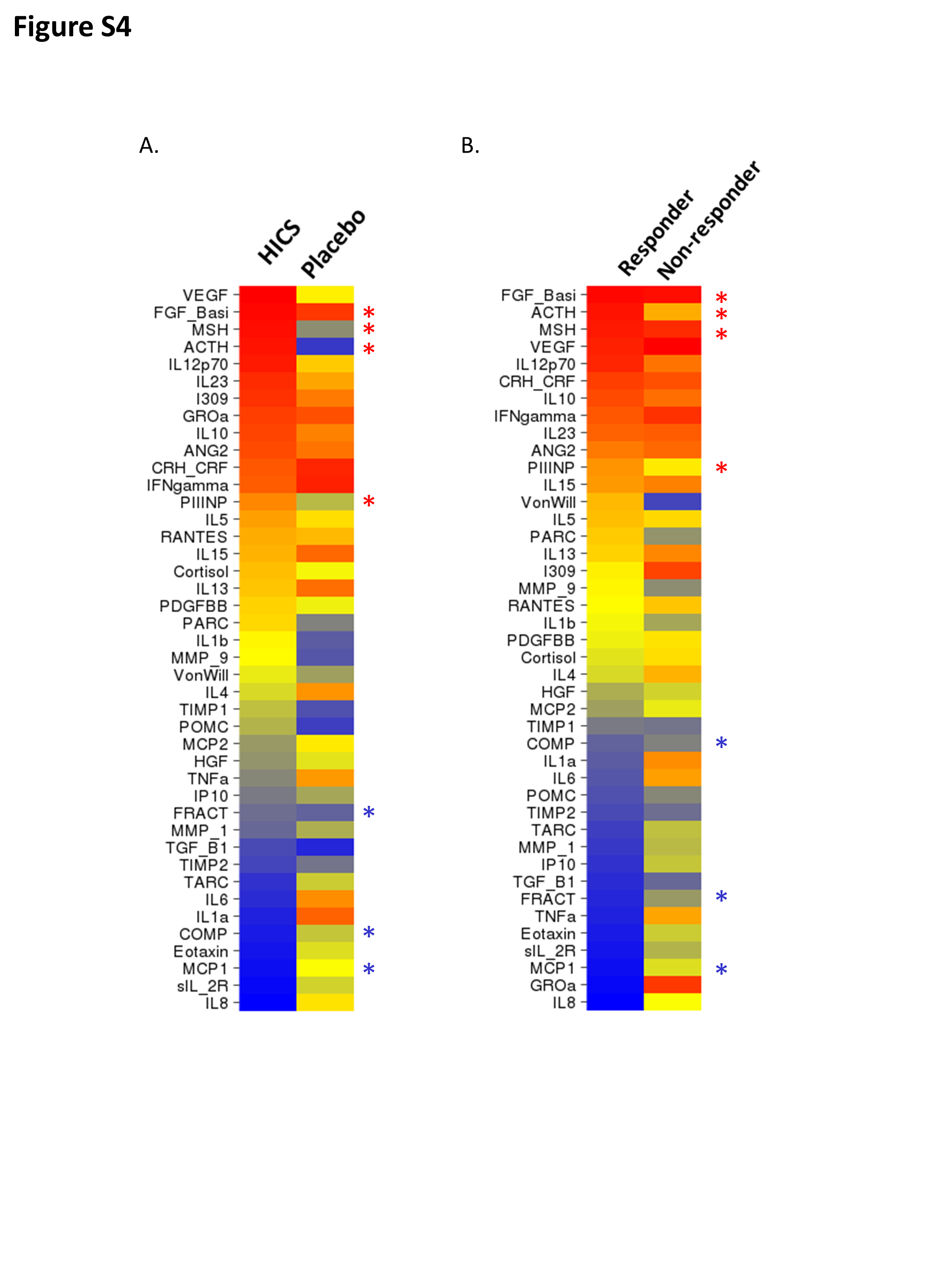

Supplement: Additional file 4: Figure S4. — Average change in serum proteins comparing treatment with hyperimmune caprine serum (HICS) or placebo and for MRSS responders versus non-responder at 26 weeks. (A) Average change in serum protein was calculated for each treatment arm over 26 weeks and ranked according to fold change average after HICS treatment. (B) Similar analysis was undertaken for average protein changes in the subjects showing significant improvement of four skin score units and 20% of baseline MRSS score during the trial (responders) or these that did not demonstrate clinical response. These were ranked for the most increased proteins in responder cases. Key proteins that emerged as upregulated (red) or downregulated (blue) for HICS treatment, shown in Fig. 4, are annotated with asterisks. (TIF 761 kb) [file 13075_2017_1252_MOESM4_ESM.tif]
